# Regional correlation of biochemical measures of amyloid and tau phosphorylation in the brain

**DOI:** 10.1186/s40478-020-01019-z

**Published:** 2020-08-27

**Authors:** Kanta Horie, Nicolas R. Barthélemy, Nipun Mallipeddi, Yan Li, Erin E. Franklin, Richard J. Perrin, Randall J. Bateman, Chihiro Sato

**Affiliations:** 1 Department of Neurology, Washington University School of Medicine, St. Louis, MO 63110 USA; 2 Department of Neurology and Division of Biostatistics, Washington University School of Medicine, St. Louis, MO 63110 USA; 3 Department of Pathology and Immunology, Washington University School of Medicine, St. Louis, MO 63110 USA; 4 Hope Center for Neurological Disorders, Washington University School of Medicine, St. Louis, MO 63110 USA; 5 Charles F. and Joanne Knight Alzheimer’s Disease Research Center, Washington University School of Medicine, St. Louis, MO 63110 USA

## Abstract

Alzheimer’s disease (AD) neuropathologic change is characterized by amyloid plaques and neurofibrillary tangles (NFTs) that consist of aggregated amyloid beta (Abeta) and hyperphosphorylated tau proteins (p-tau), respectively. Although the global relationship between Abeta and p-tau has been studied for decades, it is still unclear whether a regional correlation exists between Abeta and p-tau in the human brain. Recent studies in cerebrospinal fluid (CSF) have suggested that tau phosphorylation at specific sites such as T217 is modified at an early stage of AD when amyloid plaques become detectable. We applied biochemical and mass spectrometry methods in human brain samples with and without Abeta plaque pathology to measure site-specific phosphorylation occupancies in *soluble* and *insoluble* tau. Our quantitative results identified multiple residues specifically hyper-phosphorylated in AD, including at sites T111, T153, S184 (or S185), T205, S208, T217, S262, and S285 in brain soluble tau. In contrast, the most enriched phosphorylated residues in brain insoluble tau were T111, S113, T153, T181, S199, S202, T205, T217, T231, S262, and S396. Tau phosphorylation occupancies in the insoluble fraction were relatively constant across brain regions, suggesting that tau has a consistent phosphorylation pattern once it has aggregated into NFTs. We did not find regional association between Abeta42 and insoluble tau. However, the phosphorylation profile of soluble tau in AD brain was highly correlated to that in AD CSF, which was analyzed in a previous study. We also found a higher regional association between total Abeta42 and soluble tau phosphorylation occupancy at residues T111, T153 and T217 in the brain. This study provides insights into regional interactions between amyloidosis and specific tau phosphorylated residues in the human brain and may explain the specific increases of tau species phosphorylation observed in AD CSF.

**Electronic supplementary material:**

The online version of this article (10.1186/s40478-020-01019-z) contains supplementary material, which is available to authorized users.

## Introduction

Alzheimer’s disease (AD) is a progressive neurodegenerative disease that affects stereotyped regions of the brain and, over many years, leads to synaptic impairment and neurological decline. The neuropathological hallmarks of AD are amyloid plaques that accumulate early in AD, and neurofibrillary tangles (NFTs) that accumulate later and correlate more closely with clinical impairment. Plaques and NFTs are composed of aggregated amyloid beta (Abeta) and hyperphosphorylated tau (p-tau), respectively. A potential link and causality between Abeta and p-tau has been studied over two decades. The ‘Amyloid hypothesis’ suggests that Abeta primarily drives AD pathogenesis and that tau hyperphosphorylation, aggregation, and formation of NFTs occur downstream. However, it is still unknown whether there is a direct link between Abeta and tau hyperphosphorylation, especially on a local level in the human brain.

Recent in vivo analyses of cerebrospinal fluid (CSF) from familial AD mutation carriers have shown a close association between changes in aggregated Abeta and tau phosphorylation occupancy at specific sites [2]. Hyperphosphorylation at residue T217 increases almost simultaneously with Abeta deposition, as measured by positron emission tomography (PET), more than 20 years before symptoms onset. Other phosphorylated residues such as T181 and T205 change later in the disease trajectory, suggesting that tau phosphorylation sites are differently affected by metabolic changes subsequent to amyloidosis. Notably, all these modifications occur before the detection of tau aggregation measured by PET, concomitantly with the increase in cognitive symptoms [23], suggesting that hyperphosphorylation of CSF soluble tau occurs before substantial NFT formation. However, little is known about site specific changes in tau phosphorylation in AD brain and how it translates to CSF.

Prior work suggests high concordance between PET and CSF biomarkers for Abeta pathology [8, 33] and modest concordance between PET and CSF for tau pathology [5, 6, 14]. Such comparisons are somewhat awkward, as they relate global levels of CSF analytes to a summary of regional measures of PET. To find a direct link between Abeta and p-tau and address how local concentrations of Abeta and different p-tau species may be associated in the human brain, biochemical methods that can measure both Abeta and p-tau in the same brain tissue will be necessary. Given that soluble tau species found in CSF likely reflect soluble tau in the brain, investigating tau in brain soluble fraction would be critical. Although hyperphosphorylation sites of tau have been previously qualitatively studied in *insoluble* fractions in AD [16–18], quantitation of phosphorylation occupancy has never been annotated, especially in *soluble* fractions.

In this study, we biochemically analyzed multiple regions from human brains with and without AD neuropathologic change (ADNC) to measure local Abeta and p-tau correlation in soluble and insoluble fractions. Results from this study may provide evidence for a direct link between local Abeta and tau phosphorylation in AD. Furthermore, we combined sensitive mass spectrometry (MS) methods to extensively profile and quantitate over 20 soluble and insoluble p-tau species in human brain samples. This may provide critical insights into the interpretation of how different CSF p-tau biomarkers dynamically change in quantity and stoichiometry in AD.

## Materials and methods

### Human brain samples

Frozen postmortem brain tissue samples from cases representing different stages of ADNC were obtained from the Knight ADRC Neuropathology Core at Washington University School of Medicine. ADNC was classified according to National Institute on Aging and Alzheimer’s Association criteria [24], using data from immunohistochemistry (10D5 and PHF-1 primary antibodies) applied to formalin-fixed, paraffin-embedded tissue samples from the left side of each brain. In the first cohort, the two cases selected to represent “controls” lacked evidence of parenchymal amyloid and showed modest NFT pathology typical of older neuropathologic cohorts (Thal Abeta phase 0, Braak NFT stage II). Two cases selected to represent “early stage” AD pathology showed intermediate-to-high distributions of diffuse amyloid plaques and modest NFT pathology (these cases were: Thal Abeta phase 3, Braak NFT stage I; and Thal Abeta phase 4, Braak NFT stage II). Two cases selected to represent “late stage” AD were: Thal Abeta phase 4, Braak NFT stage V; and Thal Abeta phase 5, Braak NFT stage VI. Frozen samples from the right side of each brain were collected from 10 to 11 regions, including cerebellum (CB), superior frontal gyrus (SFG), frontal pole (FP), superior temporal gyrus (Temp), occipital lobe (Ocp), thalamus (Thal), amygdala (Amy), midbrain (Mid), pons, inferior parietal lobule (Pari), and Striatum. In the second cohort, parietal region from 20 participants with various amyloid and tau pathologies were analyzed. The study was approved by the Washington University in St. Louis Institutional Review Board, and all participants were consented for autopsy and sharing of samples.

### Fractionation of brain samples

The protocol for brain sample fractionation is described in Additional file 1: Fig. S1. Frozen brain tissue was sliced using a cryostat at − 20 °C, weighed, collected in tubes, and stored at − 80 °C prior to biochemical analyses. The tissue (300–400 mg) was sonicated in ice-cold buffer containing 25 mM tris-hydrochloride (pH 7.4), 150 mM sodium chloride, 10 mM ethylenediaminetetraacetic acid, 10 mM ethylene glycol tetraacetic acid, phosphatase inhibitor cocktail (Sigma), and protease inhibitor cocktail (Roche). Final brain tissue suspension was at a concentration of 0.3 mg/μL buffer. The tissue suspension was clarified by centrifugation for 20 min at 11,000 g at 4 °C, and the resulting supernatant was defined as “brain homogenate,” which was used for brain total (*soluble* + *insoluble*) Abeta and *soluble* tau analysis. For *insoluble* tau analysis, the brain homogenate was incubated with 1% sarkosyl for 60 min on ice, followed by ultra-centrifugation at 100,000 g at 4 °C for 60 min to obtain an insoluble pellet, which was defined as “brain insoluble extract” and used for brain *insoluble* tau analysis.

### Biochemical analysis of amyloid by MS

Brain homogenate (50 μL) containing total (*soluble* + *insoluble*) Abeta was mixed with 450 μL of phosphate-buffered saline (PBS) pH 7.4 containing 1% bovine serum albumin and 20 μL of solution containing ^15^N Abeta40 and 42 uniformly labeled (0.25 and 0.025 ng/µL, respectively. rPeptide) as an internal standard. Abeta was immunoprecipitated with HJ5.1 antibody (anti-Abeta13–28 mid domain) beads (30 μL, 3 mg/mL), then the bound Abeta was digested on-beads with 50 μL of LysN (0.25 ng/μL) in 25 mM triethyl ammonium bicarbonate (TEABC). Digests were desalted by C18 TopTip. Before eluting samples, 3% hydrogen peroxide and 3% formic acid (FA) in water was added onto the beads, followed by overnight incubation at 4 °C to oxidize the peptides containing methionine. The eluent was lyophilized and resuspended in 25 µL of 10% FA and 10% acetonitrile and 20 nM BSA tryptic digest prior to MS analysis on nanoAcquity UPLC system (Waters) coupled to Xevo TQ-S mass spectrometer (Waters) operating in selected reaction mode. Abeta40 and 42 peptides were quantified by MS.

### Insoluble tau analysis by MS

Brain insoluble extract was resuspended with 200 μL of PBS followed by sonication. The resulting suspension (10–20 μL containing 2.5 μg of total protein) was mixed with 200 μL of lysis buffer (7 M urea, 2 M thio-urea, 3% 3-[(3-cholamidopropyl)dimethylammonio]-1-propanesulfonate (CHAPS), 1.5% n-octyl glucoside, 100 mM TEABC) followed by spiking with 5 μL of solution containing ^15^N Tau-441 (2N4R) uniformly labeled (2 ng/µL, gift from Dr. Guy Lippens, Lille University, France) as an internal standard. Five μL of 500 mM dithiothreitol was added to the suspension, followed by sonication. The resulting solution was mixed with 15 μL of 500 mM iodoacetamide and incubated for 30 min at room temperature in the dark.

Protein digestion was conducted using the filter-aided sample preparation method as previously reported [25]. Briefly, each prepared solution was loaded on a Nanosep 10 K filter unit (PALL) and centrifuged. After washing the sample on the filter unit with 8 M urea in 100 mM TEABC solution, immobilized proteins were digested on the filter using endoproteinase Lys-C at 37 °C for 60 min. Then, the samples were further digested using trypsin at 37 °C overnight. The digested samples were collected by centrifugation, then desalted by C18 TopTip. In this purification process, 50 fmol each of AQUA internal-standard peptide was spiked for the quantification of some specific peptides. Before eluting samples, 3% hydrogen peroxide and 3% FA in water was added onto TopTip, followed by overnight incubation at 4 °C to oxidize the peptides containing methionine residues. The eluent was lyophilized and resuspended in 27.5 µL of 2% acetonitrile and 0.1% FA in water prior to MS analysis on nanoAcquity UPLC system coupled to Orbitrap Fusion Tribrid or Orbitrap Eclipse Tribrid mass spectrometer (Thermo Scientific) operating in PRM mode. Forty-eight brain tau peptides including 26 phosphorylation sites were measured within the insoluble tau fraction (Additional file 1: Table S1).

### Soluble tau analysis by MS

Brain homogenate (50 μL) was mixed with 450 μL of PBS and 10 μL of solution containing ^15^N Tau-441 (2N4R) uniformly labeled (200 pg/µL) as an internal standard. Brain tau isoforms were immunoprecipitated with N-terminal HJ8.5 and mid-domain Tau1 antibodies. Immunoprecipitated tau isoforms were processed and digested as described previously [26]. The same MS methods were used as described above in insoluble tau analysis section.

### Quantitative assessment of phosphorylation occupancy of tau

To assess the phosphorylation occupancy of specific sites in tau from brain homogenates and insoluble extracts, we measured the extent of phosphorylation on each site as described previously [3]. Briefly, two methods were used for this purpose: (1) phosphorylated peptides were normalized with the non-phosphorylated peptide as reference. MS/MS transition specific to each phosphorylated peptide was compared to the corresponding transition from the non-phosphorylated peptide; (2) absolute quantitation using internal synthetic labeled standards (i.e., AQUA peptides) for each phosphorylated and non-phosphorylated peptide. Signals from phosphorylated and non-phosphorylated standards were used to define an internal ratio. Because of the limited availability of AQUA peptides, a defined set of phosphorylated sites were assessed using AQUA peptides (i.e., T175, T181, S199, S202, T205, T217, T231, and S404).

## Results

### Biochemical quantitation of regional brain Abeta deposition

In the first cohort, two brains each were selected to represent control (“control”, without Abeta, and with minimal NFT pathology), early stages of ADNC (“Abeta+”, intermediate-to-high Abeta pathology and minimal NFT pathology), and late stages of ADNC (“AD’’ with advanced Abeta and NFT pathologies) (Table 1). For each case, 10 to 11 brain regions were sampled to include areas expected to harbor a broad range of amyloid plaque and NFT burdens. This cohort was used to test the hypothesis that there are regional correlations between Abeta and specific tau species. In the second cohort, parietal region from 20 participants with various Abeta and tau pathologies were analyzed (Additional file 1: Table S2). This cohort was used to confirm the results from the first cohort in one brain region across a larger number of participants.

**Table 1:** Sample demographics and neuropathological annotations in first cohort

Brains	Control		Abeta+		AD	
Participant number	#1	#2	#3	#4	#5	#6
NIA-AA score	A0B1	A0B1	A3B1	A2B1	A3B3	A3B3
Age at death	80	87	90	72	85	81
Sex	Male	Male	Male	Male	Male	Female
ApoE genotype	ε3/ε3	ε3/ε3	ε3/ε3	ε3/ε4	ε3/ε3	ε3/ε3
PMI (h)	7	47	14.5	33.3	16.3	12

NIA-AA score incorporates histopathologic assessments of Abeta deposits (A), and staging of NFTs (B) [24]. ApoE: Apolipoprotein E. PMI: postmortem interval between time of death and freezing (and beginning of fixation by immersion in formalin) of brain tissue

For each brain sample, we measured Abeta40 and 42 concentrations in corresponding brain homogenate containing both *soluble* and *insoluble* Abeta using immunoprecipitation (IP) followed by MS analysis (Additional file 1: Fig. S1). While the Abeta40 measures did not correlate with the neuropathological annotations in the second cohort samples, the Abeta42 measures were generally consistent with the neuropathological annotations (Additional file 1: Table S2), suggesting that total Abeta42 can be used as a surrogate of amyloid load. In addition, the Abeta40 concentrations were lower or equal to the Abeta42 concentrations, supporting that the Abeta species measured in this study are primarily derived from insoluble aggregates.

**Fig. 1:**
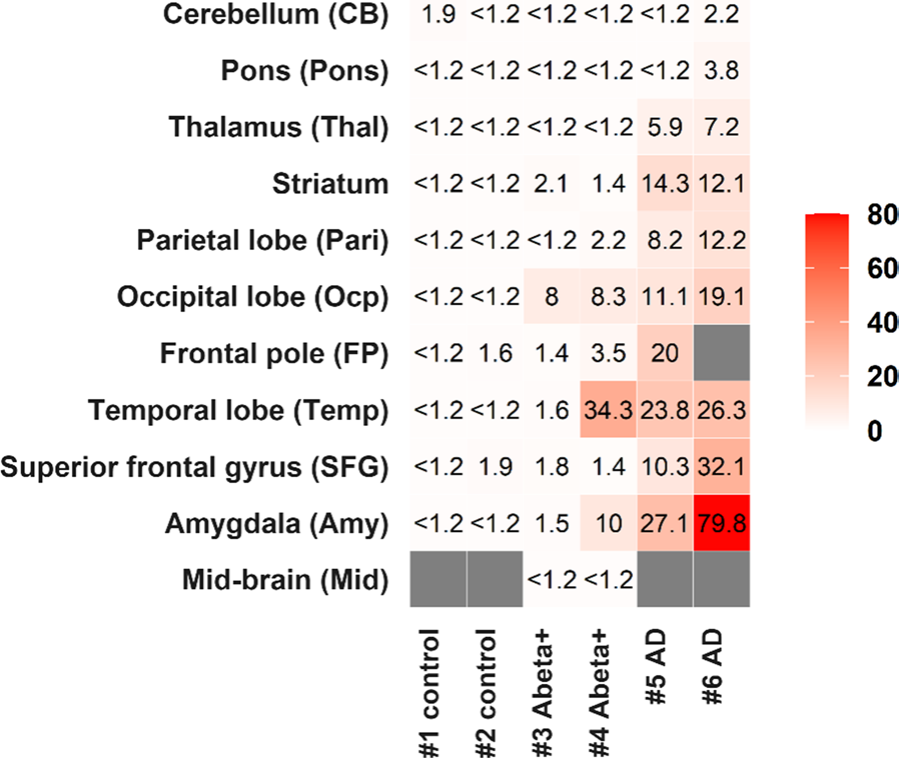
Heat map of Abeta42 concentration across different brain regions. Nine–eleven brain regions from six participants with different stages of ADNC (NIA-AA stages A0–A3 for amyloid deposition derived from Thal Abeta phases 0-5, and stages B0–B3 for Braak NFT stages 0-VI) were analyzed for Abeta42. Soluble and insoluble Abeta42 concentrations (ng/g of tissue) in brain homogenate are summarized. The data below limit of quantification (1.2 ng/g of tissue) are described as “< 1.2”. Gray: tissue was not available

In the first cohort, control cases (#1 and #2) had no quantifiable Abeta42 in homogenates from most brain areas (Fig. 1). Most brain region homogenates from AD cases had substantial amounts Abeta (SFG, FP, Temp, Ocp, Thal, Amy, Pari and Striatum). “Abeta+” brains (#3 and #4) showed intermediate Abeta42 concentrations in Ocp. The cerebellum, midbrain and pons showed less Abeta42 than the other regions, consistent with previous reports [19]. We note, however, that #4 had much higher Abeta42 concentration in Temp and Amy regions than #3; these differences may reflect Apolipoprotein E (ApoE) genotypes (i.e., #4 is ε3/ε4 carrier, Table 1), inherent variability or hemispheric asymmetry in early-stage ADNC (frozen samples are from the right hemibrain; ADNC classification was performed on the left), variations in dissection of these brain regions from case to case, and/or the potential disconnect between Thal phase (which describes anatomic distributions of amyloid plaques) and amyloid plaque density/burden, which is generally reflected by but does not directly contribute to determination of Thal phase.

### Relationship between brain MTBR-tau enrichment and Abeta

**Fig. 2:**
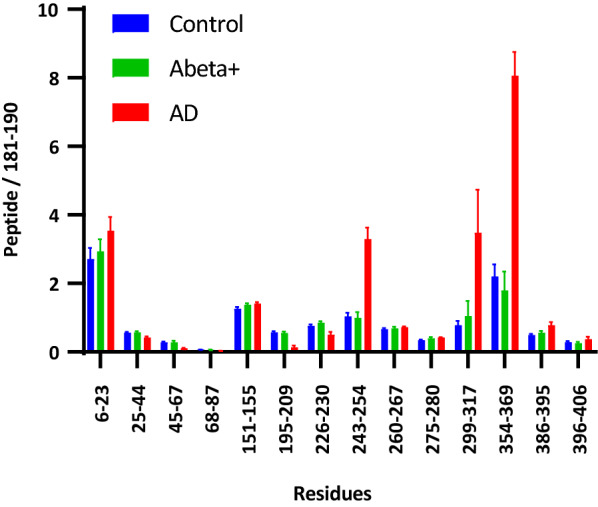
MTBR-tau is enriched in AD brain insoluble extracts in the first cohort. Enrichment profile of MTBR-tau peptides in insoluble extracts from AD (n = 15; seven or eight brain regions from two participants), Abeta+ (n = 14; seven brain regions from two participants), and control brains (n = 12; six brain regions from two participants) were analyzed by mass spectrometry. Relative abundance of tau peptides was quantified relative to mid-domain (residue 181-190) peptide for internal normalization. MTBR-tau-243 (residue 243-254), MTBR-tau-299 (residues 299-317), and MTBR-tau-354 (residues 354-369) were highly enriched in the AD brains compared to Abeta+ and controls. MTBR-tau-299 and MTBR-tau-354 are located inside the filament core of AD aggregates [12]. Unmodified residues 195-209 decreased in AD brains, potentially due to a higher phosphorylation in AD. Data are represented as mean ± SEM

A series of recent cryogenic electron microscopy (CryoEM) studies demonstrated that the core structure of tau aggregates consists of a sub-segment of the microtubule binding region (MTBR) domain and the particular conformation depends on the tauopathy [9, 10, 12, 34]. Additionally, specific enrichment of MTBR peptides over other tau peptides has been described in AD *insoluble* tau fraction [21, 25]. Therefore, we first quantified MTBR-tau in AD, Abeta+, and control brains in the first cohort to examine if MTBR-tau recapitulates tau pathology. The quantitation of 14 unmodified tau peptides in brain *insoluble* extracts confirmed that tau species containing upstream of MTBR domain (residues 243-254: MTBR-tau-243) and repeat region 2 (R2) to R3 and R4 (residues 299-317: MTBR-tau-299 and residues 354-369: MTBR-tau-354, respectively) were specifically enriched in AD compared to Abeta+ and control cases (2- to 4-fold difference in magnitude. Figure 2). This suggests that these *insoluble* MTBR-tau species recapitulate tau pathology annotated using immunohistochemistry in AD. In comparison, *soluble* tau from all brain extracts exhibited a profile consistent with full length tau as previously reported [26].

Next, we investigated if Abeta42 concentrations correlate with tau pathology measured by *insoluble* MTBR-tau species. There were low correlations between Abeta42 and *insoluble* MTBR-tau isoforms across brain regions in the first cohort (Spearman r = − 0.12, r = − 0.15, and r = − 0.03 for MTBR-tau-243, 299, and 354, respectively. Additional file 1: Fig. S2). However, the second cohort showed non-linear correlations consisting of two phases (Additional file 1: Fig. S3), which may be driven by a high number of amyloid-negative participants in the second cohort (n = 8), compared to only two cerebellum samples with no amyloid pathology in the first cohort. Nevertheless, in both cohorts, once the Abeta42 level exceeded a threshold, MTBR-tau increased in AD independently from *local* Abeta burden.

### P-tau profiles in soluble and insoluble brain fractions in AD

**Fig. 3:**
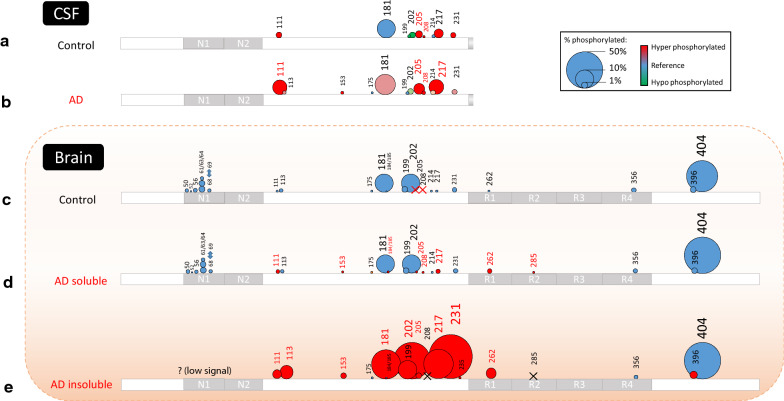
Mapping tau phosphorylation in AD brain soluble and insoluble fractions compared to CSF. Schematics of tau phosphorylation occupancies in AD CSF and brain. Tau phosphorylation occupancies in the control (**a**) and AD (**b**) CSF [3]. CSF tau is truncated after mid domain. Tau phosphorylation occupancies in control (**c**), AD soluble (**d**) and insoluble (**e**) brain fractions. T111, T153, S205 T175, S184 (or S185), S208, T217, S262, S285 are hyperphosphorylated (AD/control fold ratio > 2) in AD brain soluble tau (from brain homogenate). Phosphorylation occupancies at T111(0N), S113 (0N), T153, T181, S199, S202, T205, T217, T231, S262 and S396 are particularly increased in AD insoluble brain fractions and show a change according to the disease progression

Previously, we developed sensitive quantitative MS methods to extensively analyze p-tau species, and annotated over 20 phosphorylation sites in the CSF from individuals with AD [3]. In this study, we hypothesized that changes in p-tau species are translated from brain *soluble* fraction to CSF, and extensively profiled p-tau species comparing insoluble and soluble brain fractions from individuals with AD. Results of MS quantification are summarized in Fig. 3, Additional file 1: Table S1, and Additional file 1: Fig. S4.

In AD *insoluble* brain fractions, 14 phosphorylated sites were quantified and 11 showed pronounced increase of phosphorylation occupancy compared to control and AD soluble fractions. The list of enriched phosphorylated residues included T111, S113, T153, T181, S199, S202, T205, T217, T231, S262, and S396.

In *soluble* brain fractions, 22 and 26 phosphorylated sites were quantified in control and AD brains, respectively. We identified a pronounced increase of the phosphorylation occupancy on residues T111, T175, S184/S185 (undetermined position), T217 and S262 and new phosphorylation of residues T153, T205, S208 and S285 in AD brain soluble fraction.

We also searched for multiply phosphorylated tau peptides that would correspond to phosphorylated epitopes used for neuropathological studies (i.e., AT8 and PHF1. Additional file 1: Fig. S5) [22]. These multiply phosphorylated tau peptides were only identified in AD fractions likely due to low tau recovery from the control brain fractions, except for PHF1 (pS396 + pS404), which were identified in both control and AD soluble fractions. We also found a complex pattern of co-eluted, doubly-phosphorylated peptides for the 195-210 sequence, which differed significantly between control and AD brain extracts. After careful interpretation of LC–MS/MS signals, we attribute this differential pattern to pT205 and pS208. Phosphorylation at T205 or S208, combined with more abundant pS198, pS199 or pS202, led to these AD-associated doubly-phosphorylated peptide signals. We further observed triple phosphorylation pattern on the 195-210 peptide, but signals were too low to confidently identify the phosphorylated residues involved. This combination of phosphorylation sites could include the AT8 epitope (pS202 + pT205 + pS208). Notably, differential analysis of AD brain extracts before and after high speed centrifugation showed a decrease in the final supernatant of peptides phosphorylated at T205 and S208, suggesting their propensity for sedimentation (Additional file 1: Fig. S6).

### Local relationship between p-tau species and Abeta in AD brain

First, to test the hypothesis that *local* Abeta pathology correlates with specific p-tau species and tau aggregation in AD, we evaluated AD brain *insoluble* fractions for associations between Abeta and selected p-tau with the highest hyperphosphorylation rates (pT181, pS202, pT217, and pT231).

**Fig. 4:**
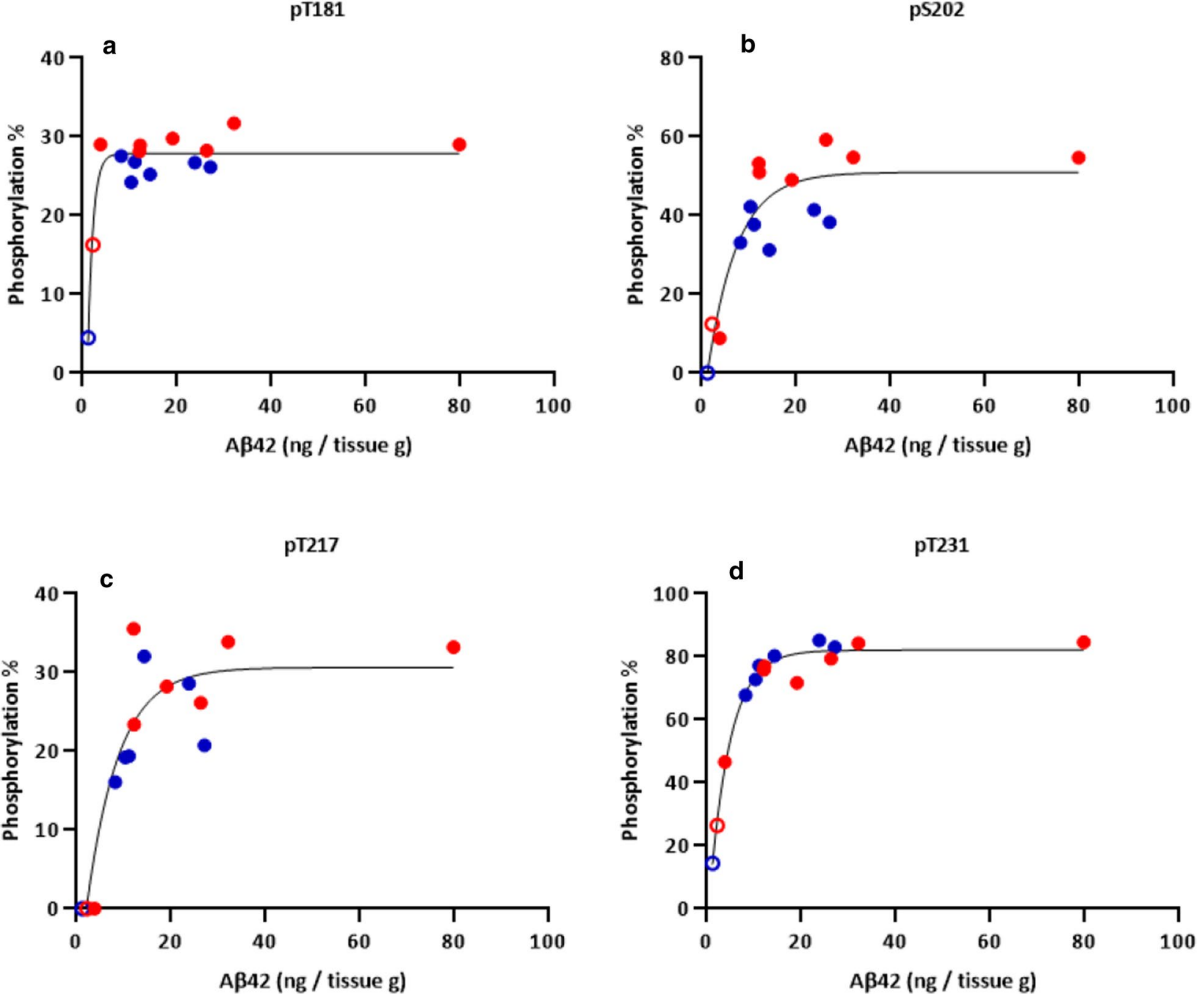
Phosphorylation occupancies of insoluble pT181, pS202, pT217, and pT231 have non-linear correlations with Abeta42 concentrations in AD brain. The phosphorylation occupancies of **a** pT181, **b** pS202, **c** pT217, and **d** pT231 in brain insoluble extracts showed non-linear correlations with soluble and insoluble Abeta42 concentrations (R^2^ = 0.90, R^2^ = 0.82, R^2^ = 0.82, and R^2^ = 0.98, respectively, in exponential-plateau model). Spearman r = 0.55, r = 0.76, r = 0.78, and r = 0.89 for pT181, pS202, pT217, and pT231, respectively. A total of 15 brain insoluble extracts from two AD participants (#5: blue, #6: red) were analyzed (regions: CB, SFG, Temp, Ocp, Amy, Pari, and Striatum from #5; CB, SFG, Temp, Ocp, Amy, Pons, Pari, and Striatum from #6). Two measures of pT217 from CB, one pT217 from Pons, and one pS202 from CB were below the detection limit and 0% were utilized in these data for modeling. Regression curves depict an exponential plateau model. Open data points indicate data from CB regions containing low amyloid pathology

In the first cohort, across the brain regions, the phosphorylation occupancy of each tau species was highly correlated with Abeta load (Spearman r = 0.55, r = 0.76, r = 0.78, and r = 0.89 for pT181, pS202, pT217, and pT231, respectively. Figure 4), suggesting that the specific p-tau species in AD NFTs may correlate regionally with local Abeta42 concentration. However, these correlations are primarily driven by the few data points from cerebellum where amyloid pathology and p-tau are both low. Indeed, the correlations between these p-tau species and Abeta may be modeled bimodally, with a very steep increase at low Abeta42 levels followed by plateauing of phosphorylation occupancies (i.e., exponential plateau curve fitting: R^2^ = 0.90 plateauing at 28% of phosphorylation occupancy, 0.82 plateauing at 51%, 0.82 plateauing at 31%, and 0.98 plateauing at 82% for pT181, pS202, pT217, and pT231, respectively). This latter model suggests that tau phosphorylation in AD NFTs are not directly associated with amyloid after an initial increase in Abeta and p-tau. In other words, an early increase in Abeta42 may be permissive for tau phosphorylation at these sites, but further increases in Abeta42 do not potentiate greater tau hyperphosphorylation in *insoluble* fraction. In the second cohort, the same correlations were confirmed except for pS202, which showed less correlation with Abeta42 (Spearman r = 0.14. Additional file 1: Fig. S7). This discrepancy may be due to the amyloid-negative group (n = 8) in the second cohort having higher concentrations of Abeta42 (7.6 ± 2.1 (SEM) ng/g of tissue) than the amyloid-negative cerebellum and pons samples in the first cohort (≤ 3.8 ng/g of tissue). This slightly high level of Abeta42 may be sufficient to induce the hyperphosphorylation of the residue S202 in *insoluble* fraction. Alternatively, parietal regions used in the second cohort may respond differently in the context of AD compared to cerebellum and pons analyzed in the first cohort.

**Fig. 5:**
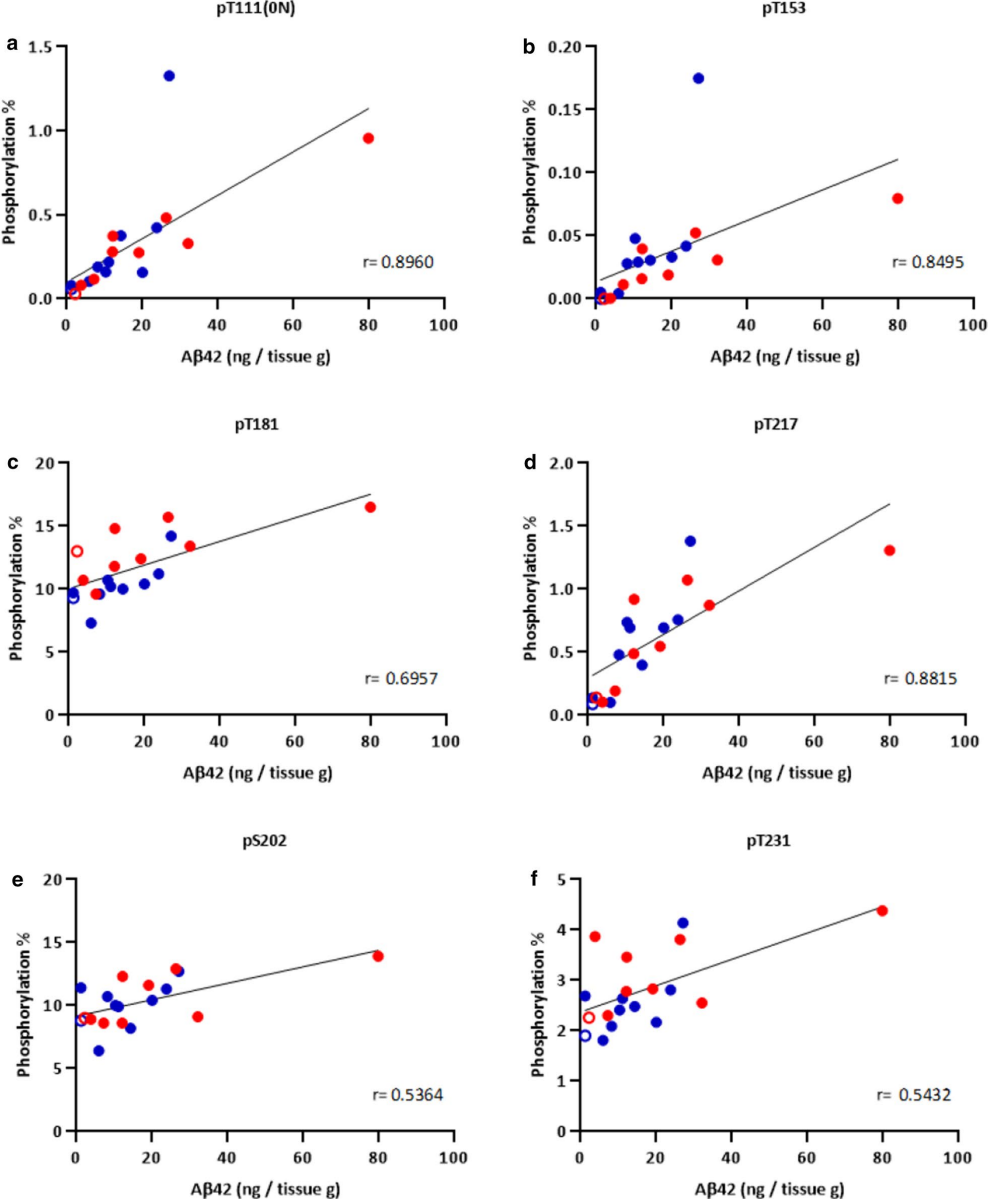
Phosphorylation occupancies of select soluble p-tau is highly and linearly correlated with Abeta42 concentrations in AD brain from the first cohort. The phosphorylation occupancies of **a** pT111 (0N), **b** pT153, **c** pT181, and **d** pT217 in brain soluble extracts showed correlations with soluble Abeta42 (Spearman r = 0.90, r = 0.85, r = 0.70, and r = 0.88, respectively). In contrast, phosphorylation at **e** pS202 and **f** pT231 were more-weakly correlated with Abeta42 (Spearman r = 0.54 and r = 0.54, respectively). A total of 19 brain soluble extracts from two AD participants (#5: blue, #6: red) were analyzed (regions: CB, SFG, FP, Temp, Ocp, Thal, Amy, Pons, Pari, and Striatum from #5; CB, SFG, Temp, Ocp, Thal, Amy, Pons, Pari, and Striatum from #6). Open data points indicate data from CB regions containing low amyloid pathology

Next, we investigated correlations between Abeta42 and these same p-tau species (pT181, pS202, pT217, and pT231) in *soluble* extracts from AD brains. In the first cohort, interestingly, pS202 and pT231 phosphorylation occupancies in *soluble* extracts showed weaker correlations with Abeta42 concentrations (Spearman r = 0.54 and r = 0.54 for pS202 and pT231, respectively. Figure 5e, f) compared to the *insoluble* extracts, suggesting that *insoluble* pS202 and pT231 are more associated with Abeta42 than *soluble* forms. In contrast, pT181 and pT217 in *soluble* fractions were more highly correlated with Abeta42 (Spearman r = 0.70, and 0.88, respectively. Figure 5c, d) than pT181 and pT217 were in *insoluble* fractions. We also examined additional p-tau species pT111 and pT153 that were only detected in soluble fractions attributed to higher MS sensitivity in soluble fractions (Fig. 5a, b). These two emerging p-tau species were also correlated with Abeta42 in *soluble* fractions to the same degree as pT217 (Spearman r = 0.90 and 0.85, respectively). Phosphorylation occupancies in the second cohort also confirmed the results in the first cohort (Additional file 1: Fig. S8). Of note, we did not observe any relationships between ApoE genotypes and Abeta42 and each p-tau species in both insoluble and soluble extracts in the second cohort (Additional file 1: Figs. S7, S8). There were no statistical differences in postmortem interval (PMI) in AD (n = 12) and control groups (n = 8). PMI did not affect phosphorylation occupancies or Abeta (data not shown).

### P-tau species are modulated in brains with Abeta pathology and modest NFT pathology

**Fig. 6:**
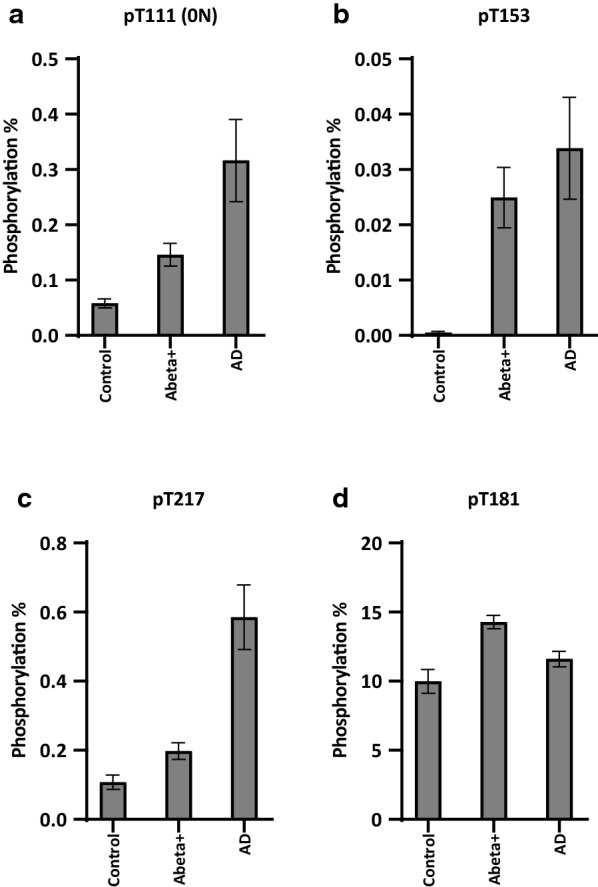
Phosphorylation occupancies of soluble pT111, pT153, and pT217 increase in AD, but pT181 does not in the first cohort. Phosphorylation occupancies at residue **a** T111 (0N), **b** T153, and **c** T217 in brain soluble extracts show staged increases in brains with Abeta pathology and with minimal tau pathology (Abeta+). Occupancies are further elevated in AD cases, in which both Abeta and tau pathology are present. **d** The phosphorylation occupancy of pT181 was approximately 10% in control brains with no clear modulation due to amyloid and/or tau pathologies. Each bar shows the mean ± SEM of two participants from each group

Next, we analyzed brain fractions from “Abeta+” cases with intermediate to high Abeta pathology and minimal NFT burden to explore whether early stage ADNC shows an intermediate correlation between Abeta and p-tau. Indeed, soluble pT111, pT153, and pT217, which are well correlated with Abeta42 (Spearman r > 0.7), showed staged elevations of phosphorylation occupancies from control to Abeta+ to AD brains in the first cohort (Fig. 6). These findings suggest that an increase in soluble p-tau at T111, T153, and T217 can occur as early as Braak NFT stage I in multiple brain areas, including areas without evidence of regional NFT pathology (by PHF-1 immunohistochemistry). The elevations of phosphorylation occupancies according to the disease progression were also confirmed in the second cohort, which showed similar staged increases across groups (amyloid negative, very mild to moderate AD, severe AD) for soluble pT111, pT153, and pT217, but not for pT181 (Additional file 1: Fig. S9).

**Fig. 7:**
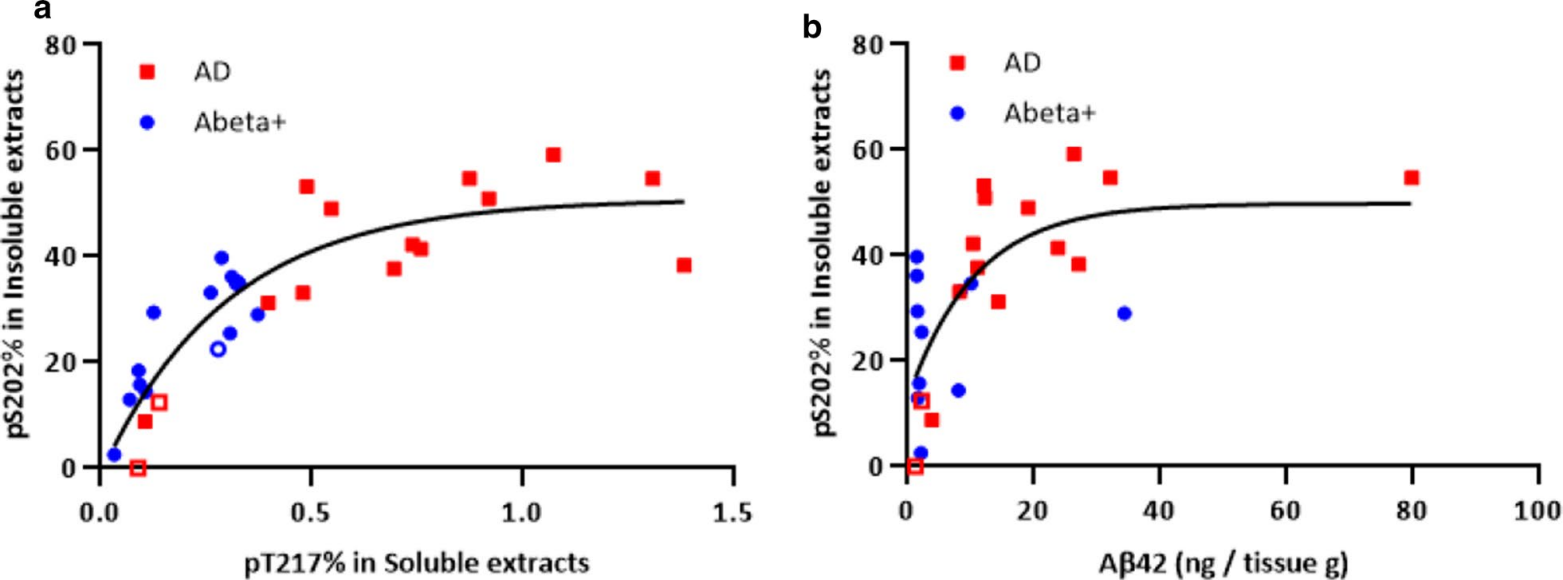
Phosphorylation occupancies of insoluble pS202 correlate more with soluble pT217 phosphorylation rates, than Abeta42. **a** Phosphorylation occupancy at T217 in brain soluble extracts showed a non-linear correlation (i.e., exponential-plateau) with pS202 in insoluble extracts (R^2^ = 0.81 plateauing at 51%). **b** Abeta 42 did not correlate with pS202% in insoluble extracts from Abeta+ brain samples (R^2^ = 0.51 plateauing at 50%). A total of 28 brain soluble- and insoluble-matched extracts were analyzed from two Abeta+ brains (regions: CB, SFG, Temp, Ocp, Amy, Pari, and Striatum from one case; CB, SFG, Temp, Ocp, Amy, Pari, and Striatum from the other participant) and two AD participants (regions: SFG, Temp, Ocp, Amy, Pari, and Striatum from one participant; CB, SFG, Temp, Ocp, Amy, Pons, Pari, and Striatum from the other case). Regression curves depict an exponential plateau model. Red squares and blue circles are individual data from AD and Abeta brains, respectively. Open data points indicate the data from CB regions containing low amyloid pathology

We observed that the phosphorylation occupancy of insoluble pS202 has a more robust correlation with the soluble pT217 rate than with Abeta42 concentration in Abeta+ and AD brains, suggesting that tau phosphorylation is more associated with tau aggregation than Abeta (Fig. 7). With continued increase of soluble pT217, insoluble pS202 reaches a plateau possibly due to the maturation of rigid NFTs composed of the most stable conformation involving specific p-tau species.

Additionally, we found variability in phosphorylation patterns for soluble pT111, pT153, and pT217 in the two Abeta+ cases in the first cohort (#3 and #4; Additional file 1: Fig. S10). Interestingly, #4 (Thal Abeta phase 3) showed global increases of the phosphorylation rates regardless of brain regions including cerebellum, and #3 (Thal Abeta phase 4) showed no changes in tau phosphorylation. It may be relevant that Abeta42 values in #4 were much higher in the temporal lobe and amygdala than in #3 (Fig. 1). These findings suggest that distribution of parenchymal Abeta plaques within the brain may not be completely predictive of increased p-tau status across brain regions during preclinical stages.

## Discussion

We used biochemical fractionation and MS methods to identify and quantify p-tau species and Abeta in AD brains with various amounts of Abeta and NFT pathologies. We demonstrated that AD brain homogenate, compared to controls, contained substantial amounts of total Abeta42 together with *soluble* p-tau species, such as pT111, pT153, pT205, pS208 and pT217. Importantly, these sites were also hyperphosphorylated in CSF tau from individuals with brain amyloidosis [2, 3], suggesting high translatability of *soluble* brain p-tau profile to CSF. We found that the extent of phosphorylation at these sites in the soluble fraction linearly associated with local Abeta, raising the possibility of a direct interplay between Abeta and *soluble* p-tau. Notably, phosphorylation occupancy on pT217 had the best association with Abeta amongst all the investigated sites, consistent with the previously described excellent correlation between amyloid PET and CSF pT217 [1, 2]. Such association would rationalize the tighter association observed between amyloid marker and CSF pT217 over other sites such as pT181. This would also support that CSF tau phosphorylation reflects *soluble* tau phosphorylation changes within the brain in response to increased Abeta.

We also showed that *insoluble* tau was phosphorylated to a much higher extent than soluble tau, especially proximal to the proline-rich region of the tau mid domain. This may support a cumulative enrichment of corresponding p-tau species in tau that form aggregates. Alternatively, tau hyperphosphorylated sites could be inaccessible to brain phosphatase due to particular tau conformation within NFTs. However, our data showing bimodal correlation between insoluble tau and Abeta would support the former. Indeed, in CSF, tau phosphorylation occupancies rapidly increase before saturating at high Abeta load, suggesting a transitional change of tau phosphorylation in parallel to Abeta deposition until reaching a final hyperphosphorylation state in mature tau aggregates. In contrast, MTBR-tau peptides that form the cores of tau aggregates in AD brains were less directly associated with Abeta, suggesting a distinct manner of aggregation and recruitment into NFTs between MTBR-tau and p-tau.

Interestingly, there was a different stoichiometry for each p-tau to be in *insoluble* versus *soluble* fraction, suggesting its propensity to be sequestered into tau aggregates. pS202 and pT231 were particularly enriched in *insoluble* fraction with phosphorylation occupancy ranging from 60 to 80% and showed lower phosphorylation occupancy in AD soluble tau, suggesting that pS202 and pT231 have higher propensity to be sequestered into *insoluble* tau. Consistently, pS202 is not found hyperphosphorylated in AD CSF and even decreases with symptoms [2, 3]. In contrast, in this study, pT217 phosphorylation occupancy was at approximately 30% in *insoluble* AD brain extracts which corresponds to an enrichment factor of about 50 times compared to *soluble* tau. However, significant hyperphosphorylation of T217 found in AD soluble brain extract and CSF tau would suggest only partial sequestration of this species into tau aggregates. T111, T205 and S262 may have similar stoichiometries. Notably, pS262 has been described as protective against tau self-aggregation [27]. However, the significant enrichment of pS262 in tau aggregates would go against such protective role. pS285, on the other hand, may be more likely to serve a protective role against aggregation as it was only detected in AD soluble tau.

Additionally, we identified multiply-phosphorylated tau species involving residues 198-199-202-205-208 in AD brain homogenates. These species are linked to the specific emergence of pT205 and pS208 in AD brain, that form the epitope that is recognized by the AT8 antibody [22], widely used in neuropathology to detect tau aggregates. Interestingly, some of these sites were also described as necessary to promote tau self-aggregation [7]. Our results would support the presence of such highly phosphorylated tau species in AD brain.

The associations between local Abeta42 and multiple soluble p-tau species shown in this study support the amyloid hypothesis; however, causality between Abeta and tau phosphorylation still needs to be addressed in future studies. Hyperphosphorylation of soluble tau may be mediated by specific kinases, reduced activity of phosphatases or result from synaptotoxicity, which may be induced by Abeta, as previously described in vitro, and in cell culture and animal models [11, 15, 30, 31]. The current study is also limited to measuring Abeta42 by MS in brain homogenate as a surrogate of Abeta pathology; this homogenate may contain a subset of *soluble* Abeta monomers, dimers and oligomers, and *insoluble* Abeta from Abeta plaques (which are more readily measured by immunohistochemistry) that became accessible to immunoprecipitation after sonication and resuspension. Potentially, only a subset of Abeta with a specific conformation may induce specific phosphorylation of soluble tau in neuronal cells. This may take place several years before tau aggregates followed by NFT formation [23].

Other limitations of this study include its use of a small number of autopsy cases within the first cohort, and variability in local correlation between Abeta and p-tau especially in the two Abeta+ cases which have different ApoE genotypes. Although we did not observe a particular effect of ApoE genotypes on Abeta or p-tau in the second cohort, this needs to be addressed in a larger cohort in a future study. Regardless, neuropathological [4] and neuroimaging [5, 20] studies using PET ligands indicate only partially overlapping spatial topologies between Abeta plaques and NFTs. This disconnect raises questions about whether *local* levels of the two proteins should be directly related, especially during preclinical stages of AD. There is growing evidence that AD pathology is strongly tied to both structural [29] and functional [13] network connectivity properties in the brain [28, 32]. This suggests that abnormal levels of tau phosphorylation may additionally be regulated by the presence of pathological Abeta in distant but *connected regions*. Focal Abeta pathology in the temporal region has been implicated in the spreading of global tau pathology, and focal Abeta pathology in the occipital region has been associated with limited spreading [28], consistent with case #4 and #3 in our study. PET ligands also measure very specific aggregated forms of the two proteins while our current results indicate that the disease process is regulating multiple soluble and insoluble tau isoforms. Biochemical assays of tissue provide a finer measure of biological processes occurring than neuroimaging but with comparatively limited spatial resolution. Future work may expand the number of brain areas sampled in an effort to elucidate the network level relationships seen with neuroimaging.

Our study provides detailed quantitative mapping of p-tau sites in AD brain and evidence that supports local interplay between Abeta and soluble p-tau. These results lay groundwork for understanding dynamic metabolism of multiple p-tau species in the brain and how it translates to CSF, assisting us with the interpretation of the changes in CSF p-tau,or potentially plasma p-tau, during AD pathogenesis.

## Supplementary Information

**Additional file 1: Figure S1.** Fractionation protocol for brain soluble and insoluble extract. Brain homogenates before sarkosyl extraction were used for total Abeta (*insoluble* + *soluble*) analyses (1) and *soluble* tau analyses (2). After sarkosyl extraction, insoluble fractions were used for *insoluble* tau analyses (3). **Figure S2**. Abeta does not correlate with MTBR-tau enrichment in AD brain insoluble extracts in the first cohort. A total of 15 brain insoluble extracts from two participants (#5: blue, #6: red) were analyzed from two AD brains (regions: CB, SFG, Temp, Ocp, Amy, Pari, and Striatum from one case; CB, SFG, Temp, Ocp, Amy, Pons, Pari, and Striatum from the other case). Open data points indicate data from cerebellum regions containing low amyloid pathology. (A) MTBR-tau-243, (B) MTBR-tau-299, and (C) MTBR-tau-354 in brain insoluble extracts showed no significant linear correlations (Spearman r = –0.12, r = –0.15, and r = –0.03, respectively) with Abeta42, suggesting that increase of MTBR-tau in AD brain is independent of Abeta pathology. However, two-phase correlations may be present in the relationship between Abeta42 and MTBR-tau in AD brain insoluble extracts, because samples from CB with less amyloid pathology showed low levels of MTBR-tau species. (D) Heat map of MTBR-tau-354 normalized enrichment across brain regions shows that there are low regional correlations with Abeta42 concentrations in Fig. 1. Gray: data was not available. **Figure S3**. Two-phase distribution may be present in the relationship between Abeta42 and MTBR-tau in AD brain insoluble extracts in the second cohort. A total of 20 brain insoluble extracts from parietal region were analyzed (n = 8 amyloid-negative (open-black), n = 12 AD). The plot colors indicate ApoE genotypes in AD brains (open-blue: ε2/ε3, open-green: ε3/ε3, open-red: ε3/ε4, filled-red: ε4/ε4). ApoE genotypes did not appear to affect the relationship between Abeta42 and MTBR-tau. (A) MTBR-tau-243, (B) MTBR-tau-299, and (C) MTBR-tau-354 in brain insoluble extracts showed significant correlations (Spearman r = 0.70, r = 0.74, and r = 0.76, respectively) with Abeta42, suggesting that increase of MTBR-tau in AD brain associates with amyloid pathology in a non-linear fashion. Presumably, once the Abeta42 level exceeded the threshold, MTBR-tau qualitatively increased, and then the correlations were no longer observed (or plateaued) in AD brain samples with high Abeta42 levels. **Figure S4**. Heat map of tau phosphorylation occupancies across different brain regions in the first cohort. Nine to eleven brain regions from six participants with different stages of ADNC (NIA-AA stages A0-A3 for amyloid deposition derived from Thal Abeta phases 0-5, and stages B0-B3 for Braak NFT stages 0-VI) were analyzed for tau phosphorylation occupancies at different sites. The data below limit of quantification are described as “ND”. Gray: data was not available. Data for insoluble pT217 (A), insoluble pT231 (B), soluble pT217 (C), and soluble pT231(D) are shown. Only tau phosphorylation occupancy in soluble pT217 (C) locally correlated with Abeta42 (described in Fig. 1). **Figure S5**. LC–MS/MS characterization of phosphorylated SGYSSPGSPGTPR (195-210) peptide from AD brain soluble fraction. (A) Mono phosphorylated peptides showing phosphorylation at residues S199, S202, T205 and S208. (B) Doubly phosphorylated peptides showing 10 combinations of phosphorylation involving residues S198, S199, S202, T205 and S208. (C) Triply phosphorylated peptides with undetermined position but at least involving pT205 + pS208. **Figure S6**. Doubly phosphorylated tau peptides from AD brain soluble fraction involving T205 and S208 disappeared after precipitation using sarkosyl extraction. MS profile of doubly phosphorylated soluble tau peptides before (A) and after (B) ultracentrifugation. The resulting soluble tau profile is similar to the profile observed in AD brain [3]. **Figure S7**. Phosphorylation occupancies of insoluble pT181, pT217, and pT231 have significant correlations with Abeta42 concentrations in AD brain from the second cohort. A total of 20 brain insoluble extracts from parietal region were analyzed (n = 8 amyloid-negative (open-black), n = 12 AD). The plot colors indicate ApoE genotypes in AD brains (open-blue: ε2/ε3, open-green: ε3/ε3, open-red: ε3/ε4, filled-red: ε4/ε4). ApoE genotypes did not appear to affect the relationship between Abeta42 and each p-tau in insoluble extracts. The phosphorylation percentages of (A) pT181, (C) pT217, and (D) pT231 in brain insoluble extracts showed significant correlations with Abeta42 concentrations (Spearman r = 0.68, r = 0.68, and r = 0.69, respectively). On the other hand, the phosphorylation percentage of (B) pS202 in brain insoluble extracts did not show any correlation with Abeta42 concentrations (Spearman r = 0.14), compared to the first cohort (Fig. 4. **Figure S8**. Phosphorylation occupancies of select soluble p-tau are significantly correlated with Abeta42 concentrations in AD brain from the second cohort. A total of 20 brain insoluble extracts from parietal region were analyzed (n = 8 amyloid-negative (open-black), n = 12 AD). The plot colors indicate ApoE genotypes in AD brains (open-blue: ε2/ε3, open-green: ε3/ε3, open-red: ε3/ε4, filled-red: ε4/ε4). ApoE genotypes did not affect the relationship between Abeta42 and each p-tau in soluble extracts. The phosphorylation occupancies of (A) pT111 (0N), (B) pT153, and (E) pT217 in brain soluble extracts showed significant correlations with soluble Abeta42 (Spearman r = 0.59, r = 0.71, and r = 0.61, respectively). Phosphorylation at (C) pT181, (D) pS202 and (F) pT231 were less correlated with Abeta42 (Spearman r = 0.36, r = 0.29 and r = 0.42, respectively), which are consistent with the results from the first cohort (Fig. 5). **Figure S9** Phosphorylation occupancies of soluble pT111, pT153, and pT217 increase in AD, but pT181 does not in the second cohort. A total of 20 brain insoluble extracts from parietal region were analyzed (n = 8 amyloid-negative (open-black), n = 5 very mild to moderate AD, n = 7 severe AD). The plot colors indicate ApoE genotypes in AD brains (open-blue: ε2/ε3, open-green: ε3/ε3, open-red: ε3/ε4, filled-red: ε4/ε4). ApoE genotypes did not show the clear differentiation for phosphorylation occupancies. Phosphorylation occupancies at residue (A) T111 (0N), (B) T153, and (C) T217 in brain soluble extracts from the second cohort showed staged increases according to AD progression. Phosphorylation occupancies are further elevated in severe AD cases, relative to very mild to moderate AD cases. (D) The phosphorylation occupancy of pT181 was approximately 10% in control brains with no clear modulation due to AD progression. These results from the second cohort are consistent with those from the first cohort (Fig. 6). Data are represented as the individual results (plots) and the mean (bar). Statistical differences were assessed with one-way ANOVA with multiple comparisons correction using Benjamini–Hochberg false discovery rate (FDR) method with FDR set at 5%. **Figure S10**. Phosphorylation occupancies of soluble pT111, pT153, and pT217 increase in AD, but pT181 does not. (Individual data from Fig. 6). Phosphorylation occupancies at residue (A) T111 (0 N), (B) T153, (C) T217, and (D) T181 in brain soluble extracts with individual data points for Fig. 6 are shown. White bars show the mean results from control (#1 and #2), Abeta+ (#3 and #4), and AD (#5 and #6) brains with individual data points representing different brain regions. Open data points indicate data from CB regions containing low Abeta pathology. **Table S1**. Summary of tau peptides analyzed in the study and phosphorylation occupancies. **Table S2**. Sample demographics and neuropathological annotations in second cohort.

## Data Availability

All data generated or analyzed during this study are included in this published article and its supplementary information.
