# Supplementary material for: Regional correlation of biochemical measures of amyloid and tau phosphorylation in the brain
Source: Acta Neuropathol Commun. 2020 Aug 27;8:149. doi: 10.1186/s40478-020-01019-z (PMC7450927; doi:10.1186/s40478-020-01019-z)
Supplement: Supplementary file 1 — Additional file 1: Figure S1. Fractionation protocol for brain soluble and insoluble extract. Brain homogenates before sarkosyl extraction were used for total Abeta (insoluble + soluble) analyses (1) and soluble tau analyses (2). After sarkosyl extraction, insoluble fractions were used for insoluble tau analyses (3). Figure S2. Abeta does not correlate with MTBR-tau enrichment in AD brain insoluble extracts in the first cohort. A total of 15 brain insoluble extracts from two participants (#5: blue, #6: red) were analyzed from two AD brains (regions: CB, SFG, Temp, Ocp, Amy, Pari, and Striatum from one case; CB, SFG, Temp, Ocp, Amy, Pons, Pari, and Striatum from the other case). Open data points indicate data from cerebellum regions containing low amyloid pathology. (A) MTBR-tau-243, (B) MTBR-tau-299, and (C) MTBR-tau-354 in brain insoluble extracts showed no significant linear correlations (Spearman r = –0.12, r = –0.15, and r = –0.03, respectively) with Abeta42, suggesting that increase of MTBR-tau in AD brain is independent of Abeta pathology. However, two-phase correlations may be present in the relationship between Abeta42 and MTBR-tau in AD brain insoluble extracts, because samples from CB with less amyloid pathology showed low levels of MTBR-tau species. (D) Heat map of MTBR-tau-354 normalized enrichment across brain regions shows that there are low regional correlations with Abeta42 concentrations in Fig. 1. Gray: data was not available. Figure S3. Two-phase distribution may be present in the relationship between Abeta42 and MTBR-tau in AD brain insoluble extracts in the second cohort. A total of 20 brain insoluble extracts from parietal region were analyzed (n = 8 amyloid-negative (open-black), n = 12 AD). The plot colors indicate ApoE genotypes in AD brains (open-blue: ε2/ε3, open-green: ε3/ε3, open-red: ε3/ε4, filled-red: ε4/ε4). ApoE genotypes did not appear to affect the relationship between Abeta42 and MTBR-tau. (A) MTBR-tau-243, (B) MTBR-tau-299, and ( [file 40478_2020_1019_MOESM1_ESM.docx]

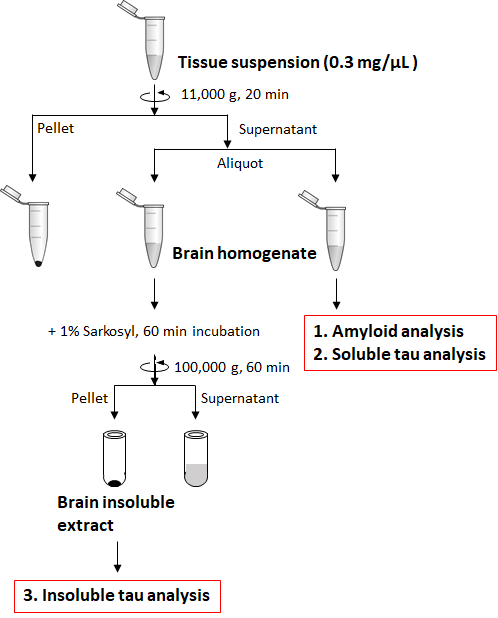


**Fig. S1. Fractionation protocol for brain soluble and insoluble extract.** Brain homogenates before sarkosyl extraction were used for total Abeta (*insoluble* + *soluble*) analyses (1) and *soluble* tau analyses (2). After sarkosyl extraction, insoluble fractions were used for *insoluble* tau analyses (3).

**Fig. S2. Abeta does not correlate with MTBR-tau enrichment in AD brain insoluble extracts in the first cohort.** A total of 15 brain insoluble extracts from two participants (#5: blue, #6: red) were analyzed from two AD brains (regions: CB, SFG, Temp, Ocp, Amy, Pari, and Striatum from one case; CB, SFG, Temp, Ocp, Amy, Pons, Pari, and Striatum from the other case). Open data points indicate data from cerebellum regions containing low amyloid pathology. (A) MTBR-tau-243, (B) MTBR-tau-299, and (C) MTBR-tau-354 in brain insoluble extracts showed no significant linear correlations (Spearman r=–0.12, r=–0.15, and r=–0.03, respectively) with Abeta42, suggesting that increase of MTBR-tau in AD brain is independent of Abeta pathology. However, two-phase correlations may be present in the relationship between Abeta42 and MTBR-tau in AD brain insoluble extracts, because samples from CB with less amyloid pathology showed low levels of MTBR-tau species. (D) Heat map of MTBR-tau-354 normalized enrichment across brain regions shows that there are low regional correlations with Abeta42 concentrations in Fig.1. Gray: data was not available.

**Fig. S3. Two-phase distribution may be present in the relationship between Abeta42 and MTBR-tau in AD brain insoluble extracts in the second cohort.** A total of 20 brain insoluble extracts from parietal region were analyzed (n=8 amyloid-negative (open-black), n=12 AD). The plot colors indicate ApoE genotypes in AD brains (open-blue: ε2/ε3, open-green: ε3/ε3, open-red: ε3/ε4, filled-red: ε4/ε4). ApoE genotypes did not appear to affect the relationship between Abeta42 and MTBR-tau. (A) MTBR-tau-243, (B) MTBR-tau-299, and (C) MTBR-tau-354 in brain insoluble extracts showed significant correlations (Spearman r=0.70, r=0.74, and r=0.76, respectively) with Abeta42, suggesting that increase of MTBR-tau in AD brain associates with amyloid pathology in a non-linear fashion. Presumably, once the Abeta42 level exceeded the threshold, MTBR-tau qualitatively increased, and then the correlations were no longer observed (or plateaued) in AD brain samples with high Abeta 42 levels.

**Fig. S4. Heat map of tau phosphorylation occupancies across different brain regions in the first cohort.** Nine to eleven brain regions from six participants with different stages of ADNC (NIA-AA stages A0-A3 for amyloid deposition derived from Thal Abeta phases 0-5, and stages B0-B3 for Braak NFT stages 0-VI) were analyzed for tau phosphorylation occupancies at different sites. The data below limit of quantification are described as “ND”. Gray: data was not available. Data for insoluble pT217 (A), insoluble pT231 (B), soluble pT217 (C), and soluble pT231(D) are shown. Only tau phosphorylation occupancy in soluble pT217 (C) locally correlated with Abeta42 (described in Fig.1).

B

A

C

**Fig. S5. LC-MS/MS characterization of phosphorylated SGYSSPGSPGTPR (195-210) peptide from AD brain soluble fraction.** (A) Mono phosphorylated peptides showing phosphorylation at residues S199, S202, T205 and S208. (B) Doubly phosphorylated peptides showing 10 combinations of phosphorylation involving residues S198, S199, S202, T205 and S208. (C) Triply phosphorylated peptides with undetermined position but at least involving pT205+pS208.

B

A

**Fig. S6. Doubly phosphorylated tau peptides from AD brain soluble fraction involving T205 and S208 disappeared after precipitation using sarkosyl extraction.** MS profile of doubly phosphorylated soluble tau peptides before (A) and after (B) ultracentrifugation. The resulting soluble tau profile is similar to the profile observed in AD brain [3].

**Fig. S7. Phosphorylation occupancies of insoluble pT181, pT217, and pT231 have significant correlations with Abeta42 concentrations in AD brain from the second cohort.** A total of 20 brain insoluble extracts from parietal region were analyzed (n=8 amyloid-negative (open-black), n=12 AD). The plot colors indicate ApoE genotypes in AD brains (open-blue: ε2/ε3, open-green: ε3/ε3, open-red: ε3/ε4, filled-red: ε4/ε4). ApoE genotypes did not appear to affect the relationship between Abeta42 and each p-tau in insoluble extracts. The phosphorylation percentages of (A) pT181, (C) pT217, and (D) pT231 in brain insoluble extracts showed significant correlations with Abeta42 concentrations (Spearman r=0.68, r=0.68, and r=0.69, respectively). On the other hand, the phosphorylation percentage of (B) pS202 in brain insoluble extracts did not show any correlation with Abeta42 concentrations (Spearman r=0.14), compared to the first cohort (Fig. 4).

**Fig. S8. Phosphorylation occupancies of select soluble p-tau are significantly correlated with Abeta42 concentrations in AD brain from the second cohort.** A total of 20 brain insoluble extracts from parietal region were analyzed (n=8 amyloid-negative (open-black), n=12 AD). The plot colors indicate ApoE genotypes in AD brains (open-blue: ε2/ε3, open-green: ε3/ε3, open-red: ε3/ε4, filled-red: ε4/ε4). ApoE genotypes did not affect the relationship between Abeta42 and each p-tau in soluble extracts. The phosphorylation occupancies of (A) pT111 (0N), (B) pT153, and (E) pT217 in brain soluble extracts showed significant correlations with soluble Abeta42 (Spearman r=0.59, r=0.71, and r=0.61, respectively). Phosphorylation at (C) pT181, (D) pS202 and (F) pT231 were less correlated with Abeta42 (Spearman r=0.36, r=0.29 and r=0.42, respectively), which are consistent with the results from the first cohort (Fig. 5).

**Fig. S9. Phosphorylation occupancies of soluble pT111, pT153, and pT217 increase in AD, but pT181 does not in the second cohort.** A total of 20 brain soluble extracts from parietal region were analyzed (n=8 amyloid-negative (open-black), n=5 very mild to moderate AD, n=7 severe AD). The plot colors indicate ApoE genotypes in AD brains (open-blue: ε2/ε3, open-green: ε3/ε3, open-red: ε3/ε4, filled-red: ε4/ε4). ApoE genotypes did not show the clear differentiation for phosphorylation occupancies. Phosphorylation occupancies at residue (A) T111 (0N), (B) T153, and (C) T217 in brain soluble extracts from the second cohort showed staged increases according to AD progression. Phosphorylation occupancies are further elevated in severe AD cases, relative to very mild to moderate AD cases. (D) The phosphorylation occupancy of pT181 was approximately 10% in control brains with no clear modulation due to AD progression. These results from the second cohort are consistent with those from the first cohort (Fig. 6). Data are represented as the individual results (plots) and the mean (bar). Statistical differences were assessed with one-way ANOVA with multiple comparisons correction using Benjamini-Hochberg false discovery rate (FDR) method with FDR set at 5%.

**Fig. S10. Phosphorylation occupancies of soluble pT111, pT153, and pT217 increase in AD, but pT181 does not. (Individual data from Fig. 6).** Phosphorylation occupancies at residue (A) T111 (0N), (B) T153, (C) T217, and (D) T181 in brain soluble extracts with individual data points for Fig. 6 are shown. White bars show the mean results from control (#1 and #2), Abeta+ (#3 and #4), and AD (#5 and #6) brains with individual data points representing different brain regions. Open data points indicate data from CB regions containing low Abeta pathology.

**Table S1. Summary of tau peptides analyzed in the study and phosphorylation occupancies.**

| **Residue** | **Peptide sequence** | **Phosphorylated site** | **Isoform** | **Phosphorylation (%)  in homogenate  (Soluble p-tau)** | | | **Phosphorylation (%)  in insoluble extract  (Insoluble p-tau)** | | | **Fold ratio  (insoluble / soluble  p-tau in AD)** | **AQUA  peptide** |
| --- | --- | --- | --- | --- | --- | --- | --- | --- | --- | --- | --- |
|  |  |  |  | **AD** | **Control** | **Fold ratio  (AD/Control)** | **AD** | **Control** | **Fold ratio  (AD/Control)** |  |  |
| 45-67 | ESPLQpTPTEDGSEEPGSETSDAK | T50 | 1N/2N | 0.33 | 0.33 | 1.0 | NQ | NQ | N/A | N/A | − |
| 45-67 | ESPLQTPpTEDGSEEPGSETSDAK | T52 | 1N/2N | 0.10 | 0.07 | 1.4 | NQ | NQ | N/A | N/A | − |
| 45-67 | ESPLQTPTEDGpSEEPGSETSDAK | S56 | 1N/2N | 0.26 | 0.20 | 1.3 | NQ | NQ | N/A | N/A | − |
| 45-67 | ESPLQTPTEDGSEEPGSETSDAK + mono-phospho | S61 or T63 or S64% (site A) | 1N/2N | 0.48 | 0.31 | 1.5 | NQ | NQ | N/A | N/A | − |
| 45-67 | ESPLQTPTEDGSEEPGSETSDAK + mono-phospho | S61 or T63 or S64% (site B) | 1N/2N | 0.70 | 0.83 | 0.8 | NQ | NQ | N/A | N/A | − |
| 45-67 | ESPLQTPTEDGSEEPGSETSDAK + mono-phospho | S61 or T63 or S64% (site C) | 1N/2N | 1.1 | 0.70 | 1.6 | NQ | NQ | N/A | N/A | − |
| 68-126 | STPTAEAEEAGIGDpTPSLEDEAAGHVTQAR | T111 (1N) | 1N | 0.07 | 0.02 | 3.5 | NQ | NQ | N/A | N/A | − |
| 68-126 | STPTAEAEEAGIGDTPpSLEDEAAGHVTQAR | S113 (1N) | 1N | 0.42 | 0.33 | 1.3 | NQ | NQ | N/A | N/A | − |
| 103-126 | AEEAGIGDpTPSLEDEAAGHVTQAR | T111 (0N) | 0N | 0.32 | 0.06 | 5.3 | 1.40 | NQ | N/A | 4.4 | − |
| 103-126 | AEEAGIGDTPpSLEDEAAGHVTQAR | S113 (0N) | 0N | 1.9 | 0.62 | 3.1 | 5.27 | NQ | N/A | 2.8 | − |
| 151-155 | IApTPR | T153 | all | 0.03 | NQ | N/A | 0.57 | NQ | N/A | 19.0 | + |
| 171-180 | IPAKpTPPAPK | T175 | all | 0.14 | 0.07 | 2.0 | 0.26 | NQ | N/A | 1.9 | + |
| 175-190 | TPPAPKpTPPSSGEPPK | T181 | all | 11.6 | 9.99 | 1.2 | 25.5 | 14.5 | 1.8 | 2.2 | + |
| 181-190 | TPPSSGEPPK + mono-phosphorylation | S184 or S185 | all | 0.17 | 0.013 | >10 | NQ | NQ | N/A | N/A | − |
| 195-209 | SGYSpSPGSPGTPGSR | S199 | all | 1.1 | 0.81 | 1.3 | 14.6 | 3.2 | 4.6 | 13.5 | + |
| 195-209 | SGYSSPGpSPGTPGSR | S202 | all | 10.2 | 7.78 | 1.3 | 40.5 | 18.7 | 2.2 | 4.0 | + |
| 195-209 | SGYSSPGSPGpTPGSR | T205 | all | 0.15 | NQ | N/A | 1.77 | NQ | N/A | 11.8 | + |
| 195-209 | SGYSSPGSPGTPGpSR | S208 | all | 0.17 | NQ | N/A | NQ | NQ | N/A | N/A | − |
| 212-221 | TPpSLPTPPTR | S214 | all | 0.05 | 0.03 | 1.7 | NQ | NQ | N/A | N/A | − |
| 212-221 | TPSLPpTPPTR | T217 | all | 0.58 | 0.11 | 5.3 | 26.4 | NQ | N/A | 45.5 | + |
| 226-234 | VAVVRpTPPK | T231 | all | 2.8 | 1.95 | 1.4 | 68.5 | 26.0 | 2.6 | 24.4 | + |
| 260-267 | IGpSTENLK | S262 | all | 0.39 | 0.007 | >10 | 3.1 | 1.7 | 1.9 | 8.0 | − |
| 282-290 | LDLpSNVQSK | S285 | 4R | 0.07 | NQ | N/A | NQ | NQ | N/A | N/A | − |
| 354-369 | IGpSLDNITHVPGGGNK | S356 | all | 0.40 | 0.36 | 1.1 | 0.01 | NQ | N/A | 0.03 | − |
| 396-406 | pSPVVSGDTSPR | S396 | all | 0.30 | 0.19 | 1.6 | 0.59 | NQ | N/A | 2.0 | − |
| 396-406 | SPVVSGDTpSPR | S404 | all | 52.2 | 34.2 | 1.5 | 57.1 | 34.6 | 1.7 | 1.1 | + |
| 6-23 | QEFEVMEDHAGTYGLGDR | No phosphorylation | all | N/A | | | | | | | − |
| 25-44 | DQGGYTMHQDQEGDTDAGLK | No phosphorylation | all |  |  |  |  |  |  |  | − |
| 45-67 | ESPLQTPTEDGSEEPGSETSDAK | No phosphorylation | 1N/2N |  |  |  |  |  |  |  | − |
| 68-87 | STPTAEDVTAPLVDEGAPGK | No phosphorylation | 1N/2N |  |  |  |  |  |  |  | − |
| 68-126 | STPTAEAEEAGIGDTPSLEDEAAGHVTQAR | No phosphorylation | 1N |  |  |  |  |  |  |  | − |
| 103-126 | AEEAGIGDTPSLEDEAAGHVTQAR | No phosphorylation | 0N |  |  |  |  |  |  |  | − |
| 88-126 | QAAAQPHTEIPEGTTAEEAGIGDTPSLEDEAAGHVTQAR | No phosphorylation | 1N/2N |  |  |  |  |  |  |  | − |
| 151-155 | IATPR | No phosphorylation | all |  |  |  |  |  |  |  | − |
| 175-180 | TPPAPK | No phosphorylation | all |  |  |  |  |  |  |  | − |
| 181-190 | TPPSSGEPPK | No phosphorylation | all |  |  |  |  |  |  |  | + |
| 195-209 | SGYSSPGSPGTPGSR | No phosphorylation | all |  |  |  |  |  |  |  | + |
| 212-221 | TPSLPTPPTR | No phosphorylation | all |  |  |  |  |  |  |  | + |
| 226-230 | VAVVR | No phosphorylation | all |  |  |  |  |  |  |  | + |
| 243-254 | LQTAPVPMPDLK | No phosphorylation | all |  |  |  |  |  |  |  | − |
| 260-267 | IGSTENLK | No phosphorylation | all |  |  |  |  |  |  |  | − |
| 275-280 | VQIINK | No phosphorylation | 4R |  |  |  |  |  |  |  | − |
| 282-290 | LDLSNVQSK | No phosphorylation | 4R |  |  |  |  |  |  |  | + |
| 299-317 | HVPGGGSVQIVYKPVDLSK | No phosphorylation | 4R |  |  |  |  |  |  |  | − |
| 354-369 | IGSLDNITHVPGGGNK | No phosphorylation | all |  |  |  |  |  |  |  | + |
| 386-395 | TDHGAEIVYK | No phosphorylation | all |  |  |  |  |  |  |  | − |
| 396-406 | SPVVSGDTSPR | No phosphorylation | all |  |  |  |  |  |  |  | + |
| 407-438 | HLSNVSSTGSIDMVDSPQLATLADEVSASLAK | No phosphorylation | all |  |  |  |  |  |  |  | − |

Phosphorylation (%) is calculated as an average of all brain regions from two AD or control participants. N/A: not applicable. NQ: Not quantifiable due to low signal.

**Table S2. Sample demographics and neuropathological annotations in second cohort.**

| **Participant number** | **Amyloid status** | **NIA-AA score** | **Age at death** | **Sex** | **ApoE genotype** | **PMI (hour)** | **Brain Abeta42  (ng/ tissue g)** | **Brain Abeta40 (ng/ tissue g)** | **CDR** | **Clinical group** |
| --- | --- | --- | --- | --- | --- | --- | --- | --- | --- | --- |
| 1 | negative | A1B0 | 72 | female | ε3ε3 | 15 | 2.2 | 18.9 | 0 | Amyloid (-) |
| 2 | negative | A1B1 | 79 | female | ε3ε3 | 50 | 3.8 | BLQ | 0 | Amyloid (-) |
| 3 | negative | A1B1 | 79 | female | ε3ε3 | 25 | 4.1 | 19.0 | 0.5 | Amyloid (-) |
| 4 | negative | A2B1 | 83 | male | ε3ε3 | 15.3 | 5.7 | BLQ | 1 | Amyloid (-) |
| 5 | negative | A0B1 | 86 | male | ε2ε3 | 13 | 2.7 | 19.4 | 2 | Amyloid (-) |
| 6 | negative | A2B1 | 91 | male | ε2ε3 | 5.5 | 17.8 | BLQ | 2 | Amyloid (-) |
| 7 | negative | A2B3 | 93 | male | ε3ε3 | 41 | 14.7 | BLQ | 3 | Amyloid (-) |
| 8 | negative | A-B2 | 78 | female | ε3ε4 | 4 | 9.6 | 19.9 | 3 | Amyloid (-) |
| 9 | positive | A3B3 | 78 | female | ε3ε4 | 25 | 73.3 | 46.9 | 0.5 | Very mild AD |
| 10 | positive | A3B3 | 89 | male | ε3ε4 | 9 | 135.7 | 23.2 | 1 | Mild AD |
| 11 | positive | A-B3 | 73 | male | ε3ε4 | 4.5 | 79.3 | 21.0 | 1 | Mild AD |
| 12 | positive | A-B3 | 89 | male | ε3ε3 | 15 | 74.9 | 24.4 | 1 | Mild AD |
| 13 | positive | A-B2 | 79 | male | ε3ε4 | 23.5 | 50.3 | 23.9 | 2 | Moderate AD |
| 14 | positive | A3B3 | 69 | male | ε2ε3 | 13.1 | 59.5 | 24.5 | 3 | Severe AD |
| 15 | positive | A3B3 | 85 | male | ε3ε4 | 5.3 | 93.7 | 42.6 | 3 | Severe AD |
| 16 | positive | A-B3 | 86 | male | ε4ε4 | 23 | 92.1 | 81.3 | 3 | Severe AD |
| 17 | positive | A-B3 | 88 | male | ε3ε4 | 8.5 | 108.2 | 100.1 | 3 | Severe AD |
| 18 | positive | A-B3 | 77 | male | ε3ε3 | 19.5 | 82.3 | 23.0 | 3 | Severe AD |
| 19 | positive | A3B3 | 86 | male | ε3ε3 | 16.3 | 34.6 | 20.5 | 3 | Severe AD |
| 20 | positive | A3B3 | 77 | male | ε3ε4 | 28 | 33.2 | 21.4 | 3 | Severe AD |

Amyloid status was defined by intra-vitam CSF Abeta42/40, neuropathology, and brain Abeta42 concentrations. NIA-AA score incorporates immunohistochemical assessments of parenchymal Abeta deposits (A) and NFT stage (B) [24]. The cases with "A-" do not have Thal Abeta phase data available for precise A score assignment. PMI: postmortem interval (hours) between time of death and freezing (and beginning of fixation by immersion in formalin) of brain tissue. BLQ: below limit of quantification (<18.1 ng/ tissue g), CDR: clinical dementia rating.
